# Supplementary material for: The impact of non-pharmaceutical interventions on the first COVID-19 epidemic wave in South Africa
Source: BMC Public Health. 2023 Aug 5;23:1492. doi: 10.1186/s12889-023-16162-0 (PMC10403893; doi:10.1186/s12889-023-16162-0)
Supplement: Supplementary file 1 — Additional file 1. [file 12889_2023_16162_MOESM1_ESM.docx]

**Appendix:**


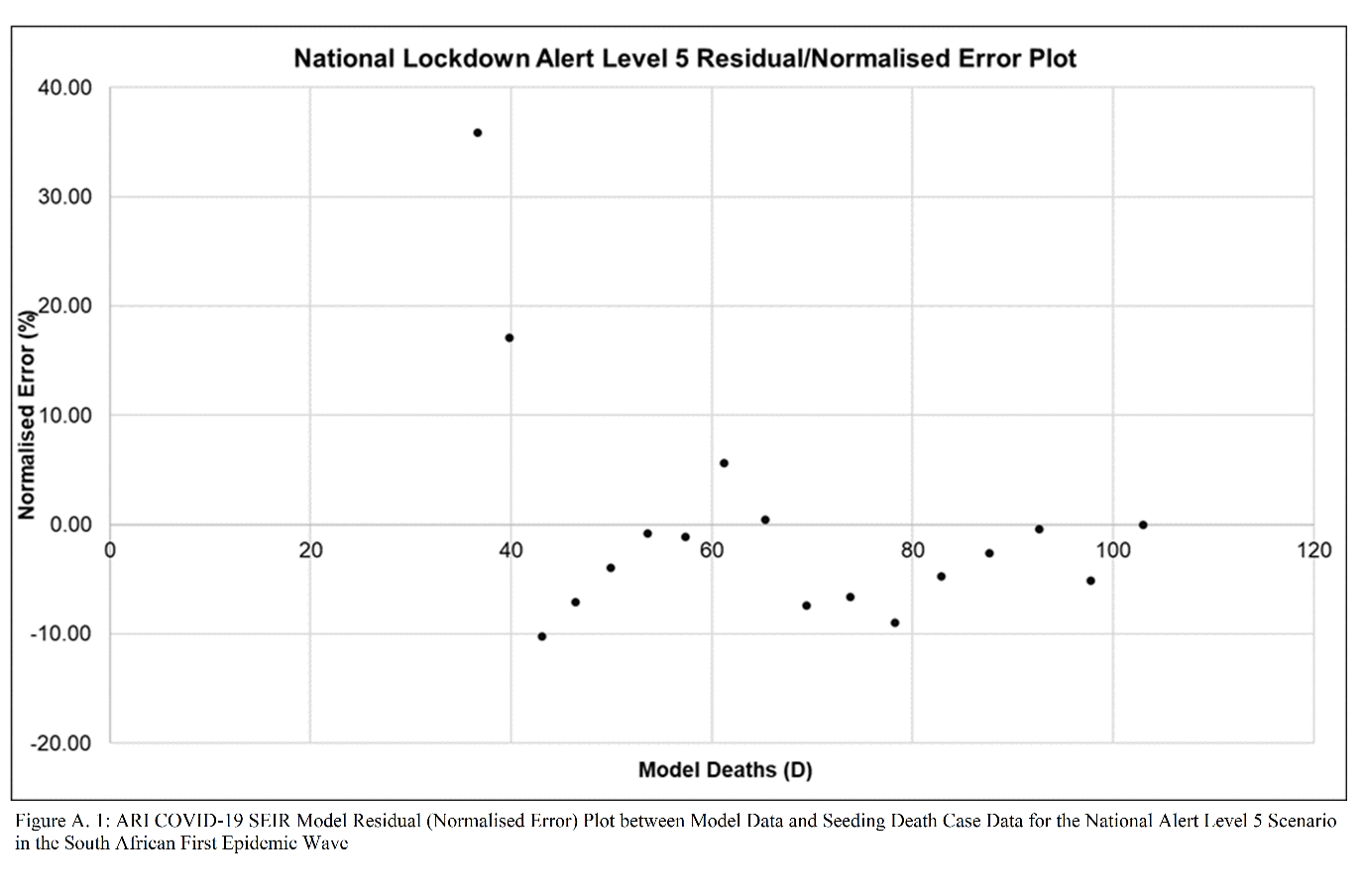


*Figure A. 1: ARI COVID-19 SEIR Model Residual (Normalised Error) Plot between Model Data and Seeding Death Case Data for the National Alert Level 5 Scenario in the South African First COVID-19 Epidemic Wave*


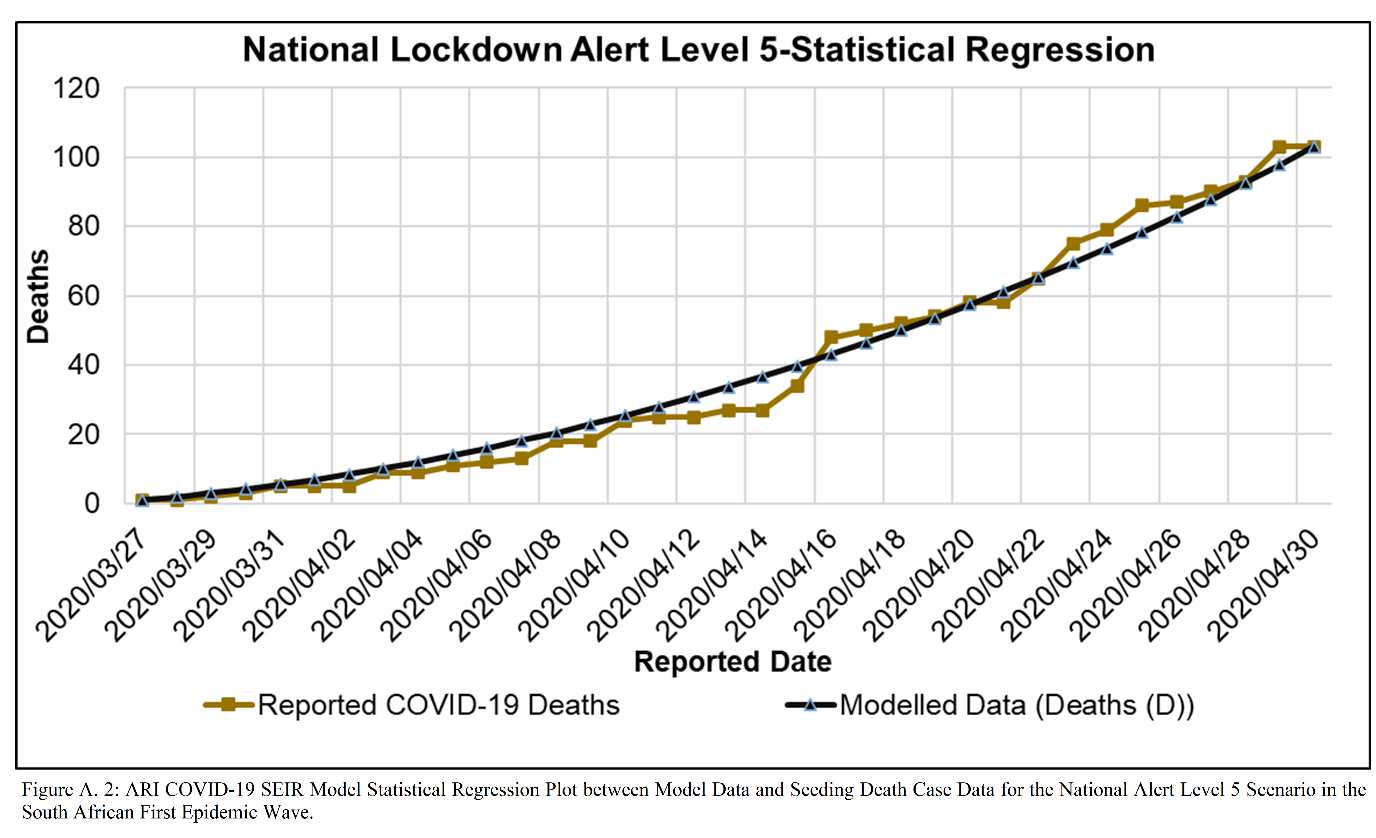


Figure A. 2: ARI COVID-19 SEIR Model Statistical Regression Plot between Model Data and Seeding Death Case Data for the National Alert Level 5 Scenario in the South African First COVID-19 Epidemic Wave.


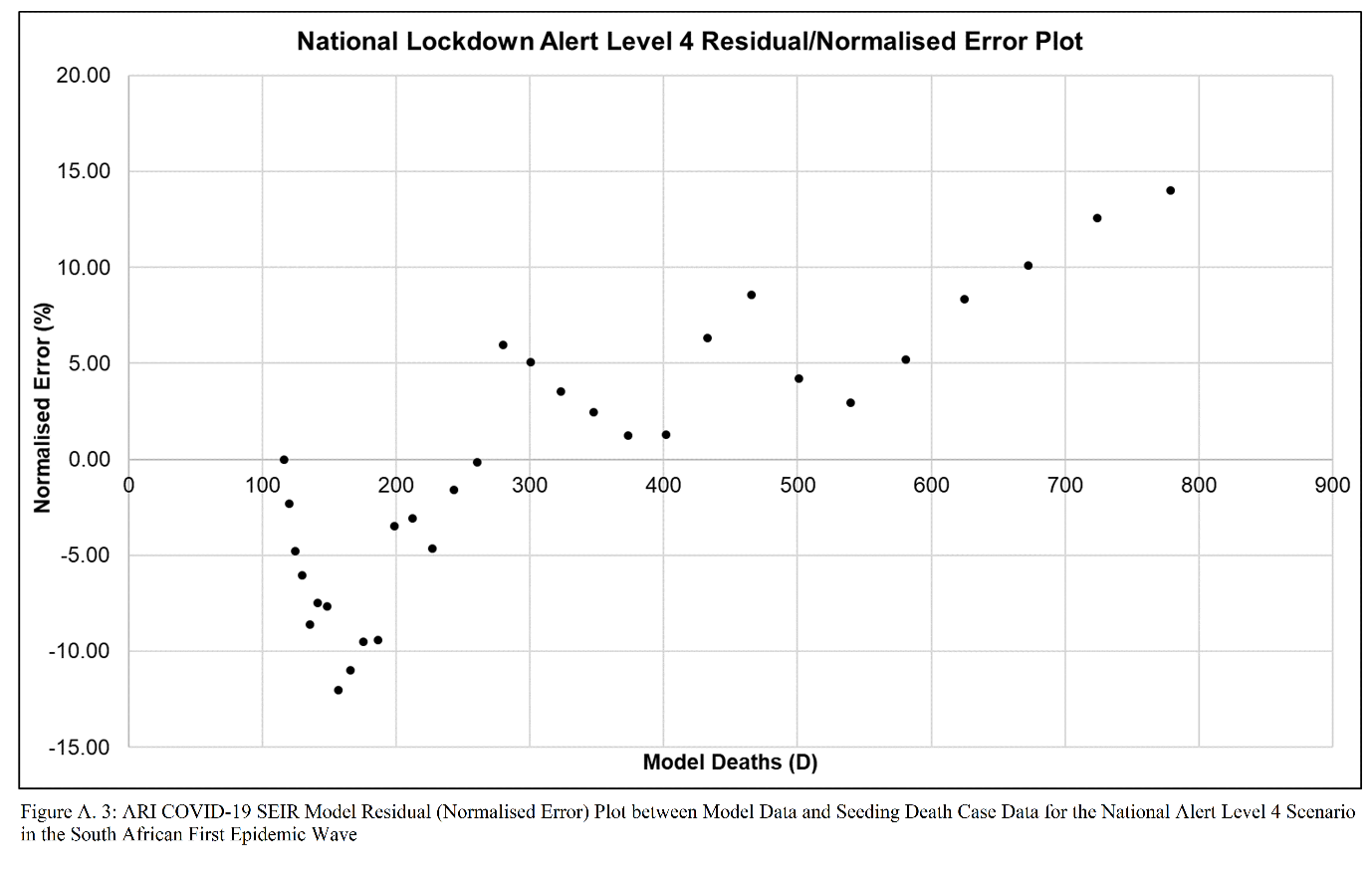


Figure A. 3: *ARI COVID-19 SEIR Model Residual (Normalised Error) Plot between Model Data and Seeding Death Case Data for the National Alert Level 4 Scenario in the South African First COVID-19 Epidemic Wave*


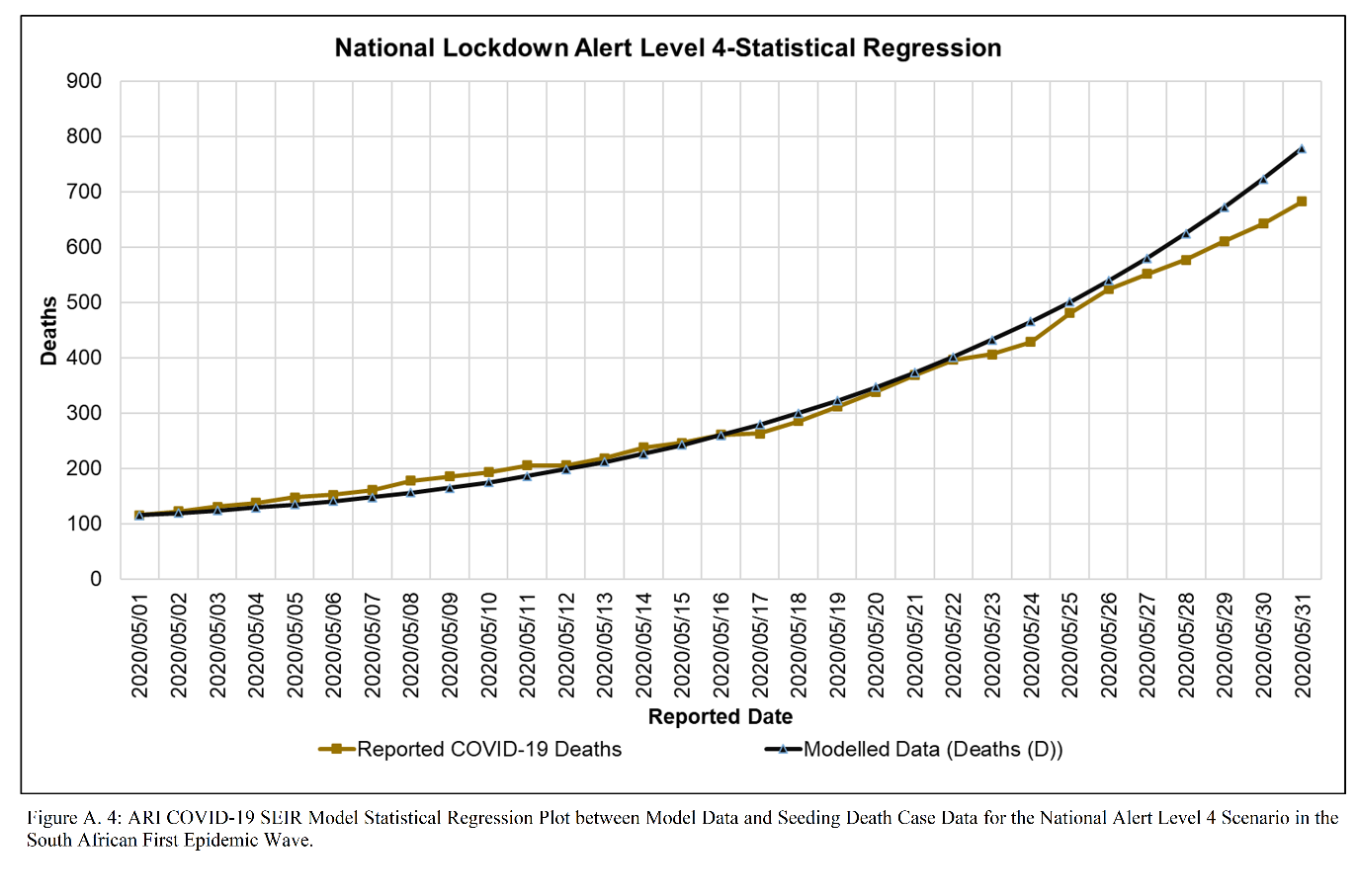


Figure A. 4: ARI COVID-19 SEIR Model Statistical Regression Plot between Model Data and Seeding Death Case Data for the National Alert Level 4 Scenario in the South African First COVID-19 Epidemic Wave.


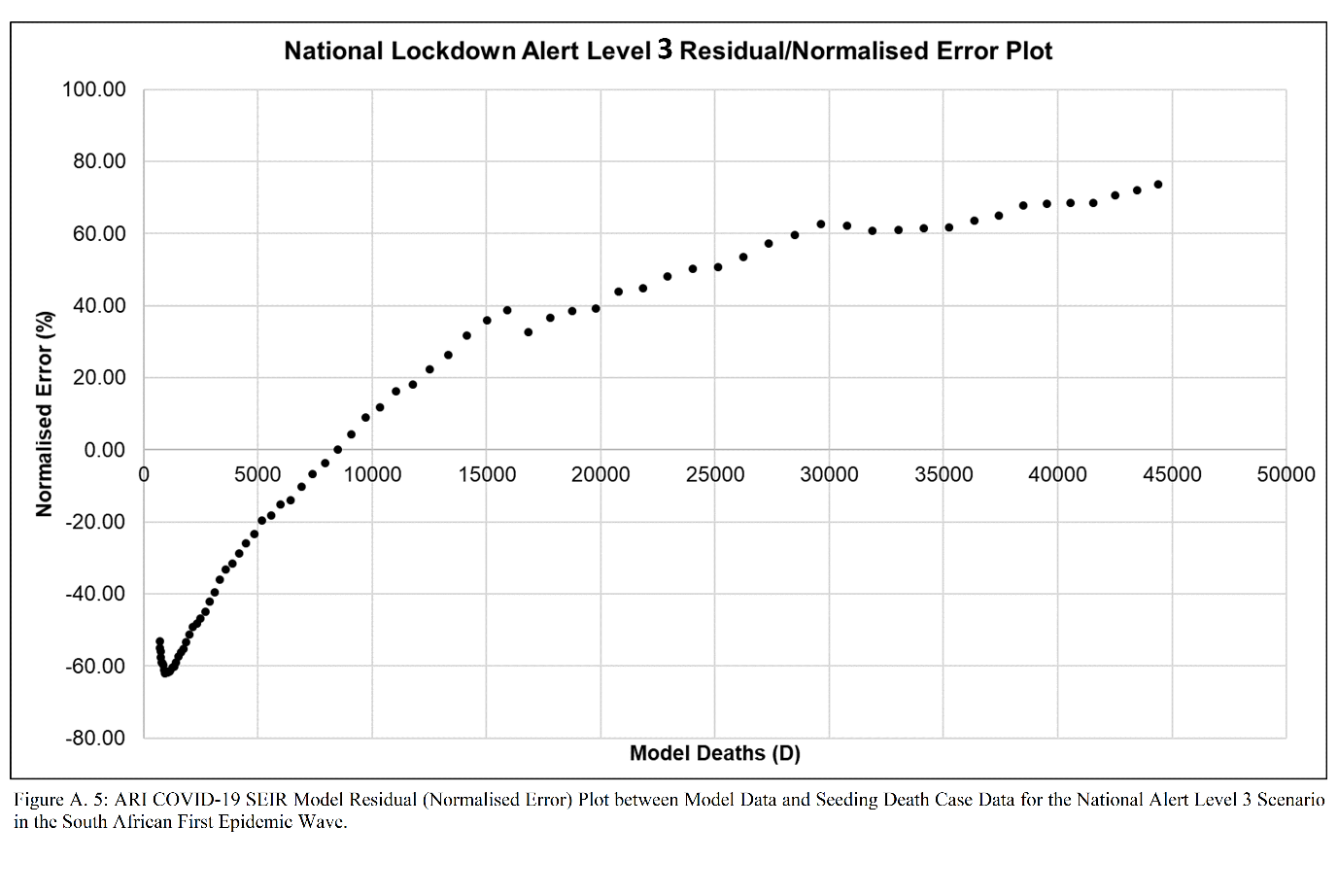


Figure A. 5: *ARI COVID-19 SEIR Model Residual (Normalised Error) Plot between Model Data and Seeding Death Case Data for the National Alert Level 3 Scenario in the South African First COVID-19 Epidemic Wave*


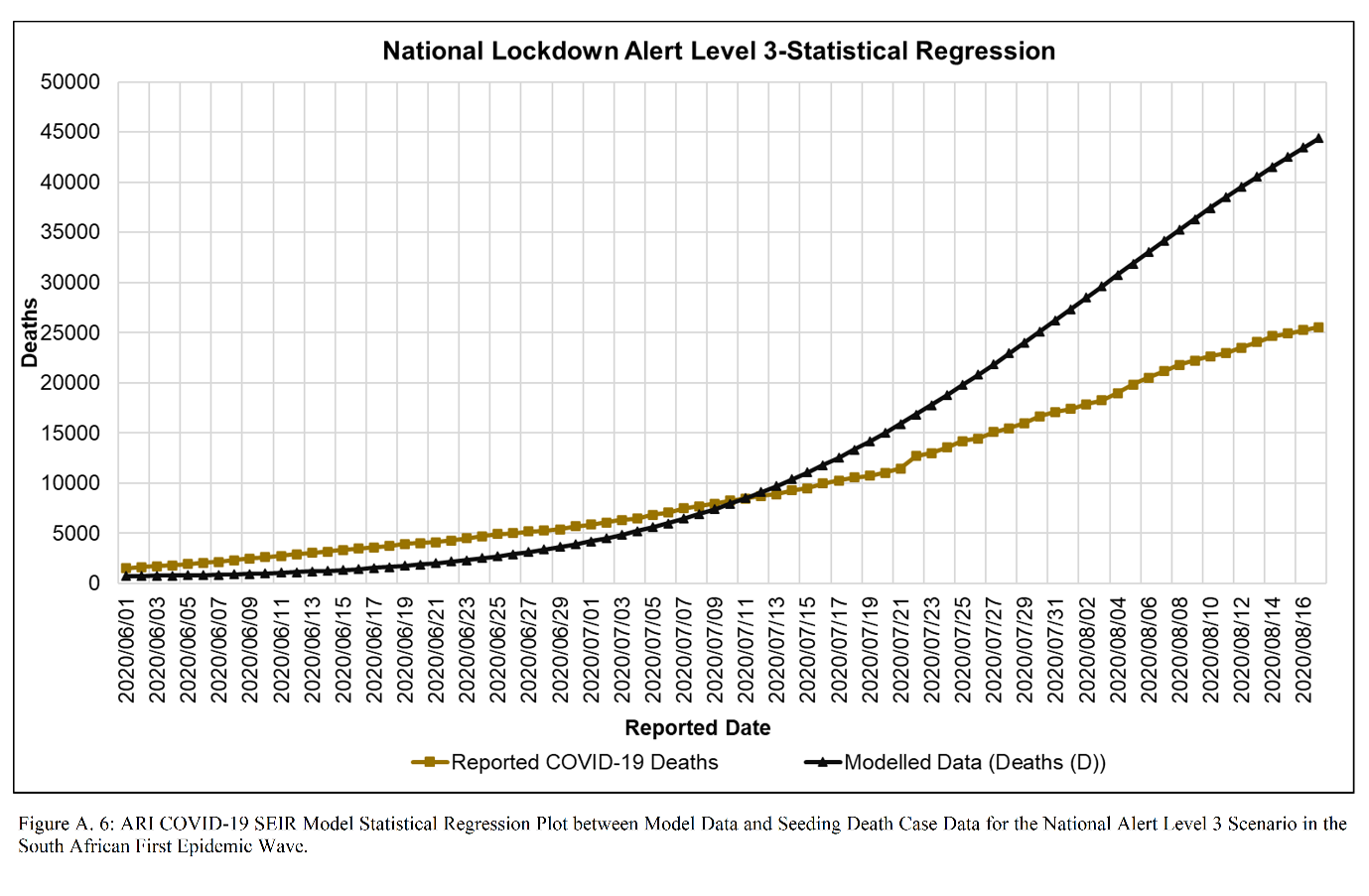


Figure A. 6: ARI COVID-19 SEIR Model Statistical Regression Plot between Model Data and Seeding Death Case Data for the National Alert Level 3 Scenario in the South African First COVID-19 Epidemic Wave.


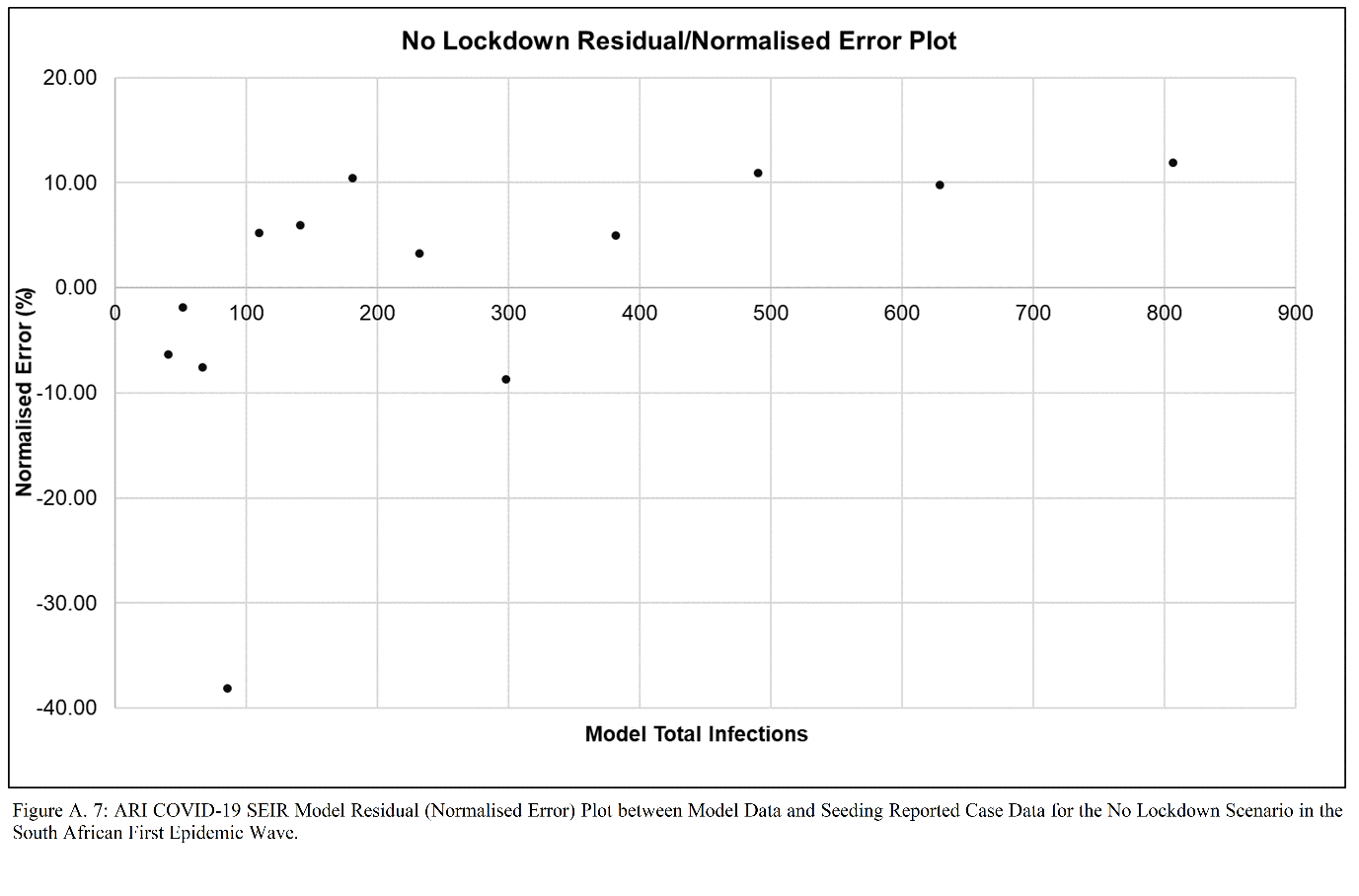


Figure A. 7: *ARI COVID-19 SEIR Model Residual (Normalised Error) Plot between Model Data and Seeding Death Case Data for the No Lockdown Scenario in the South African First COVID-19 Epidemic Wave*


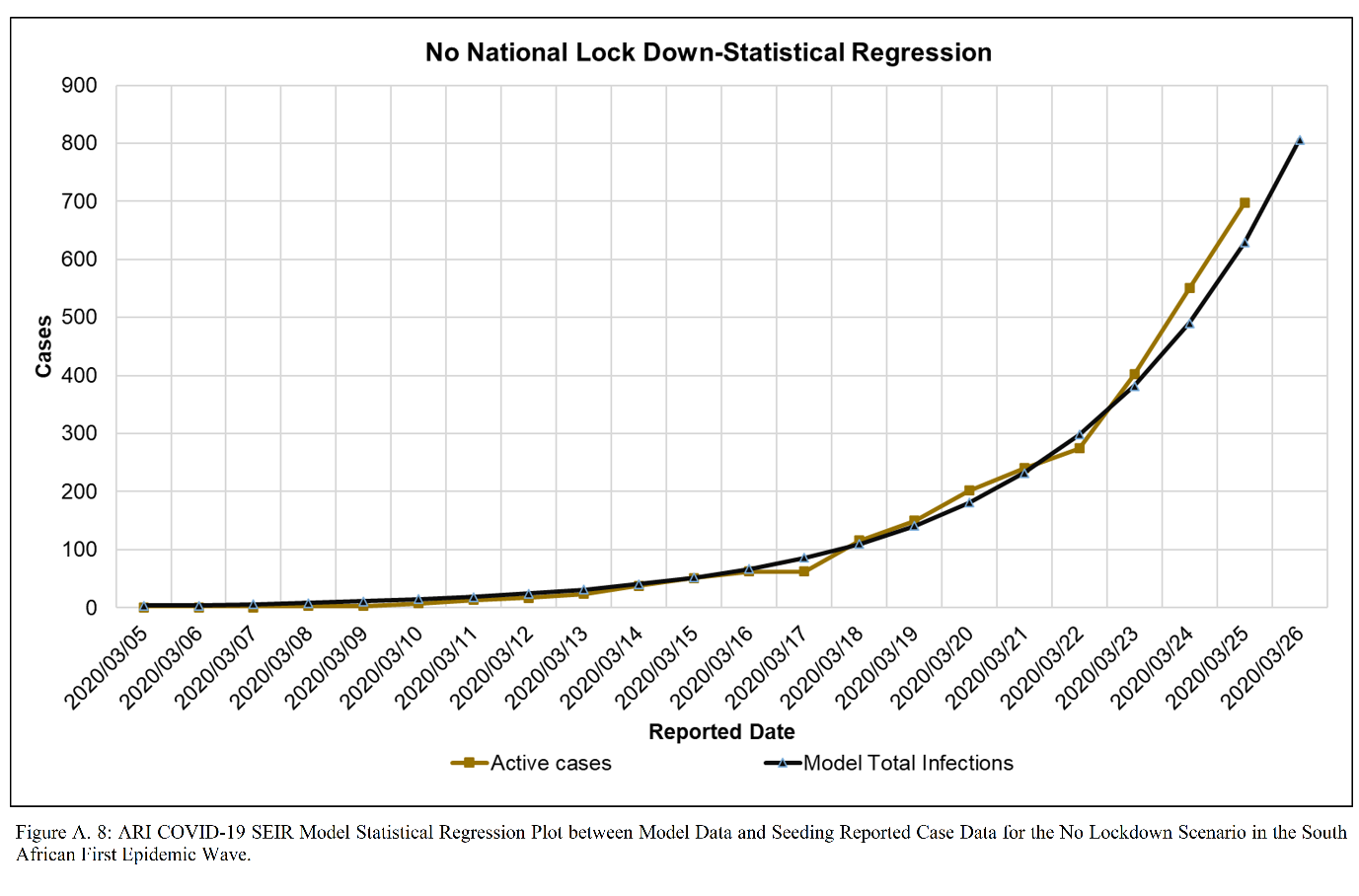


Figure A. 8: ARI COVID-19 SEIR Model Statistical Regression Plot between Model Data and Seeding Death Case Data for the No Lockdown Scenario in the South African First COVID-19 Epidemic Wave.


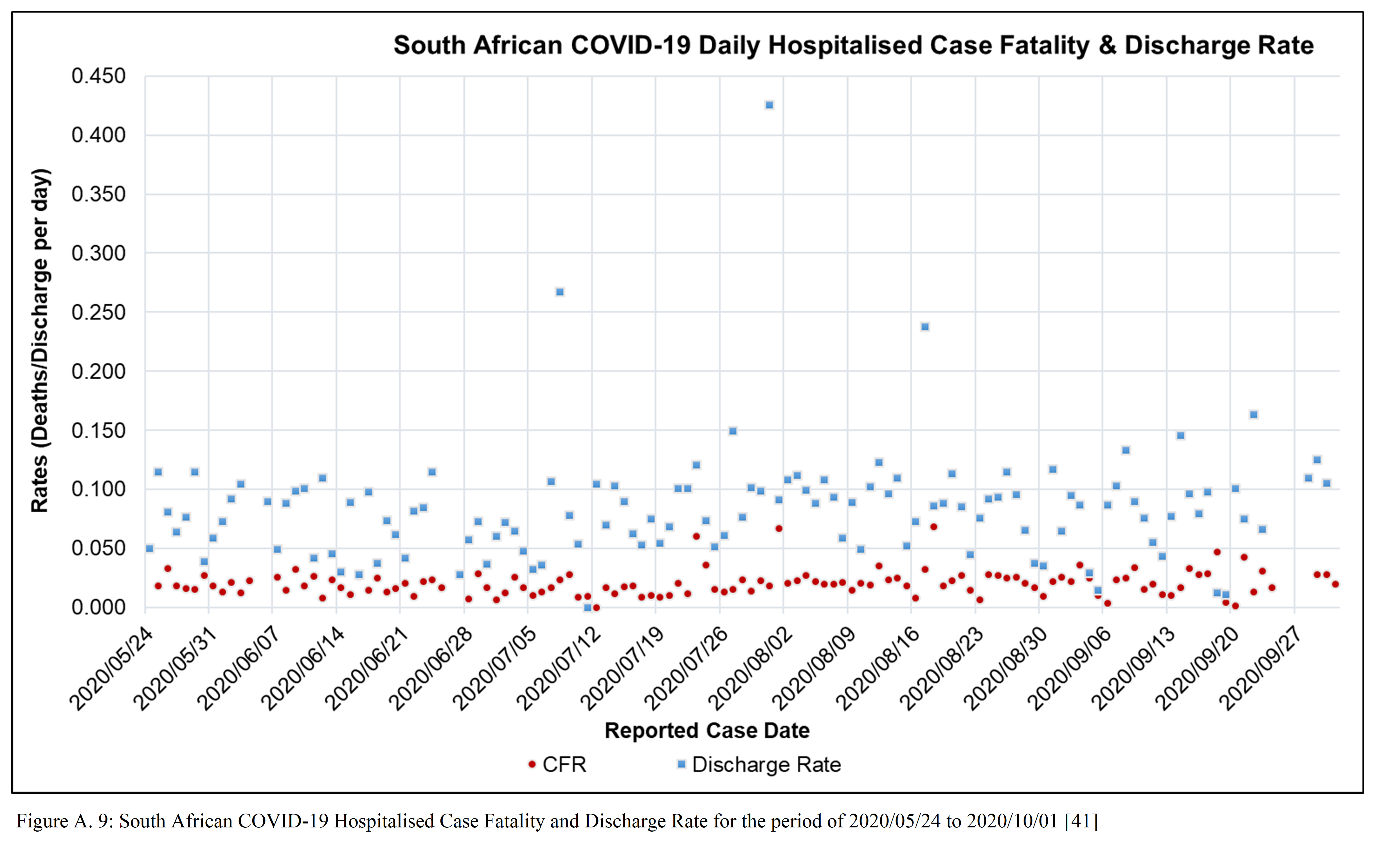


Figure A. 9: South African COVID-19 Hospitalised Case Fatality and Discharge Rate for the period of 2020/05/24 to 2020/10/01 [41]
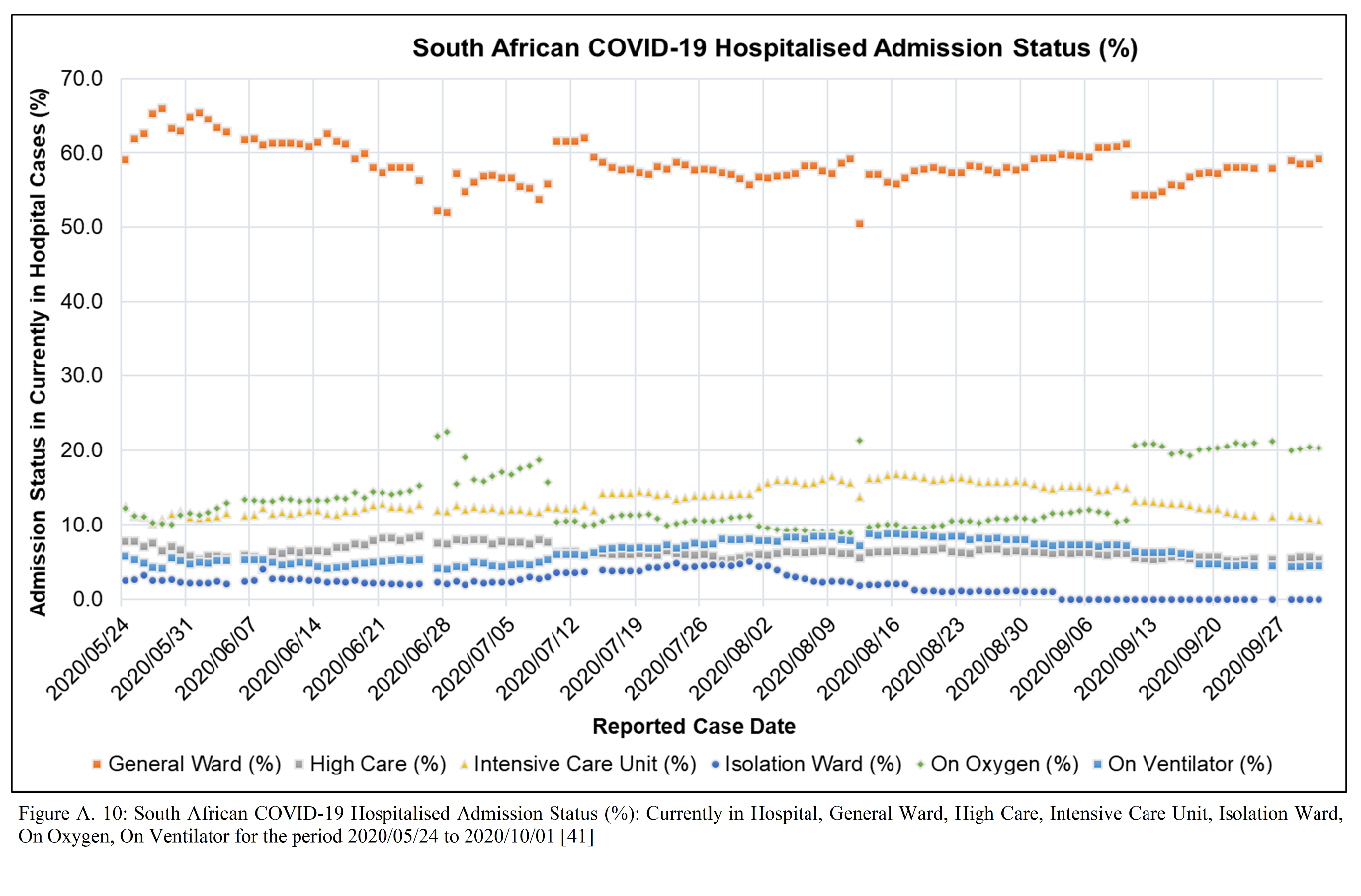


Figure A. 10: South African COVID-19 Hospitalised Admission Status (%): Currently in Hospital, General Ward, High Care, Intensive Care Unit, Isolation Ward, On Oxygen, On Ventilator for the period 2020/05/24 to 2020/10/01 [41]


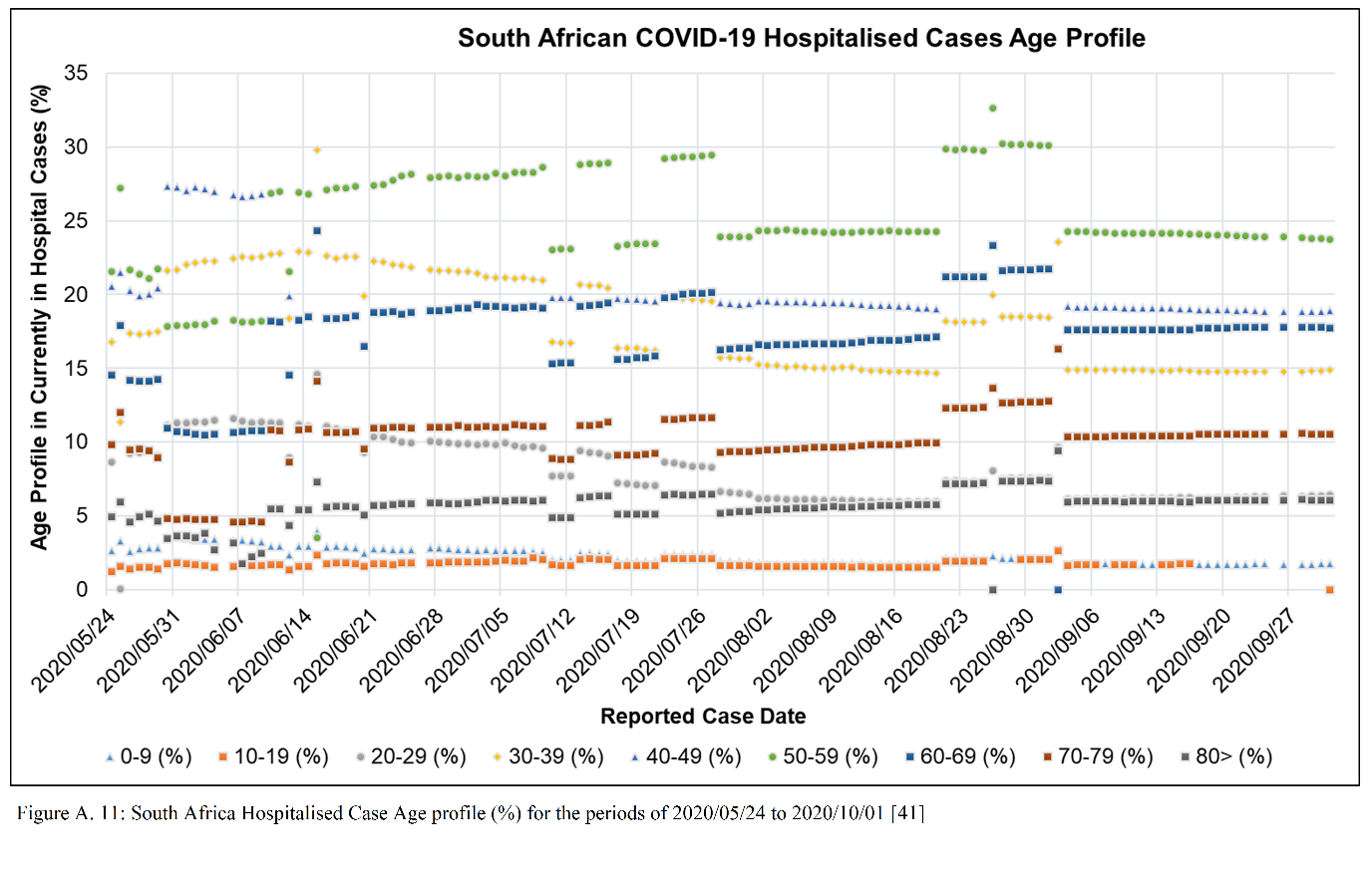


Figure A. 11: South Africa Hospitalised Case Age profile (%) for the period of 2020/05/24 to 2020/10/01 [41]


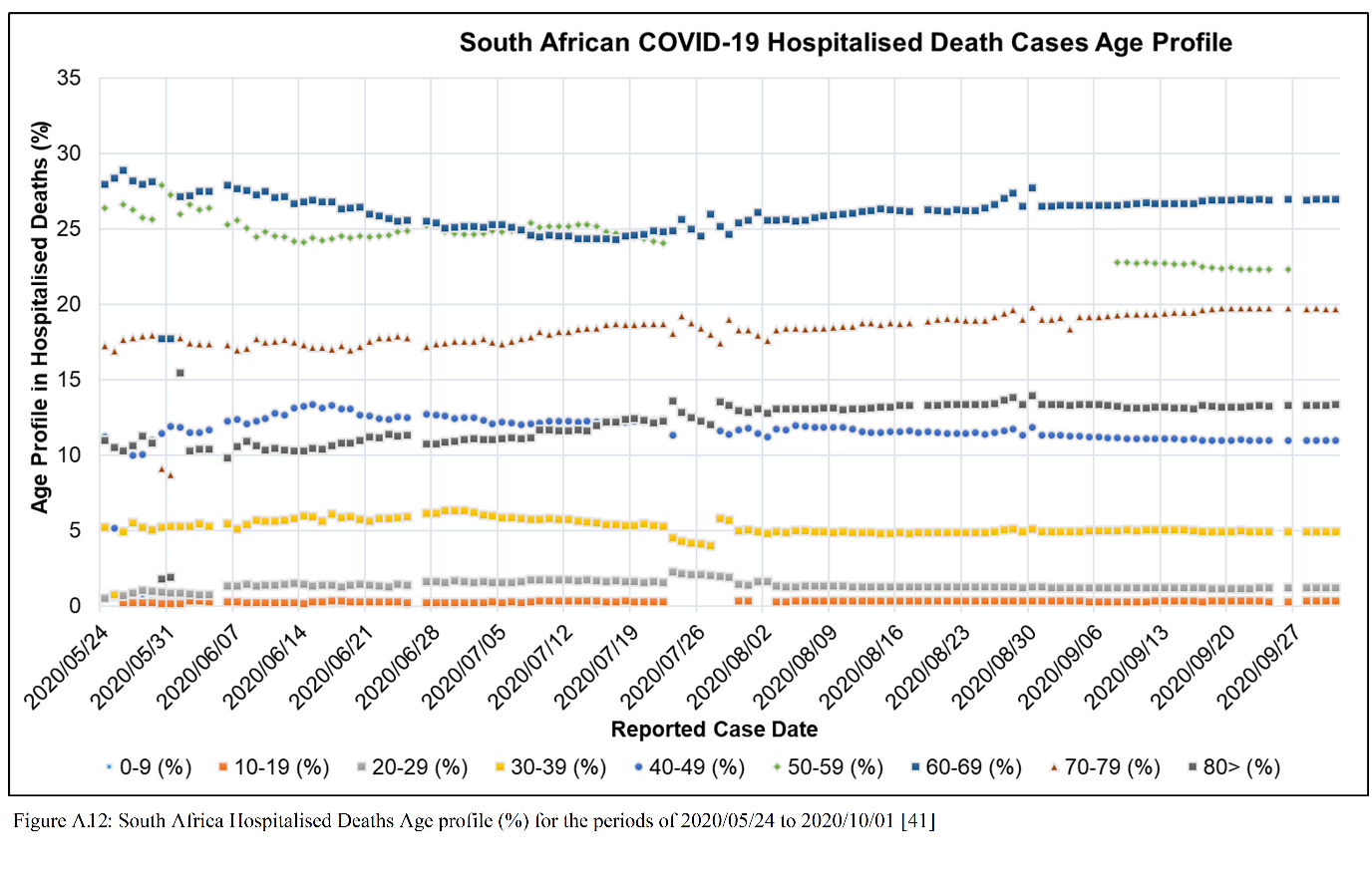


Figure A. 12: South Africa Hospitalised Deaths Age profile (%) for the period of 2020/05/24 to 2020/10/01 [41]


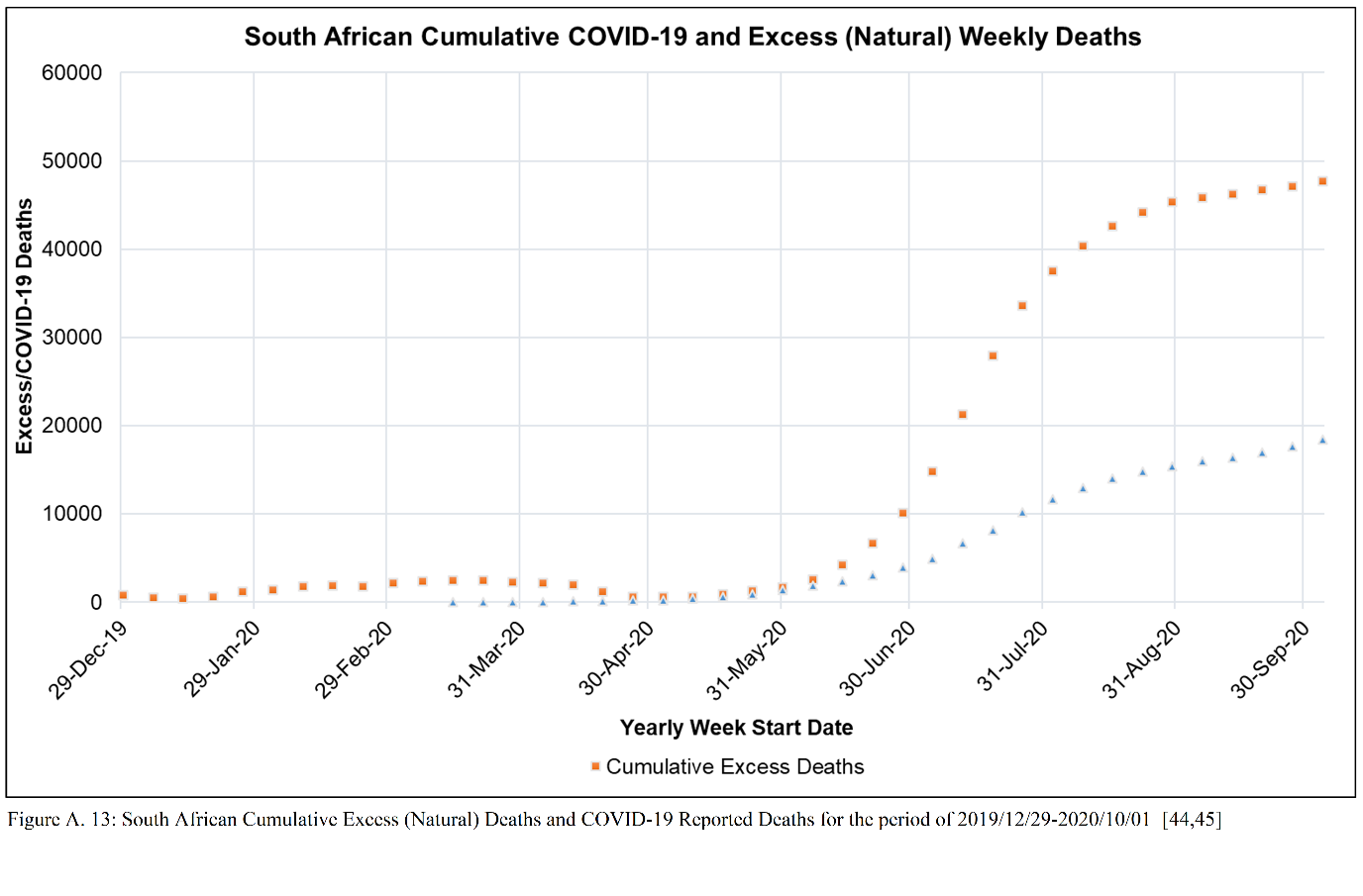


Figure A. 13: South African Cumulative Excess (Natural) Deaths and COVID-19 Reported Deaths for the period of 2019/12/29 to 2020/10/01 [44,45]


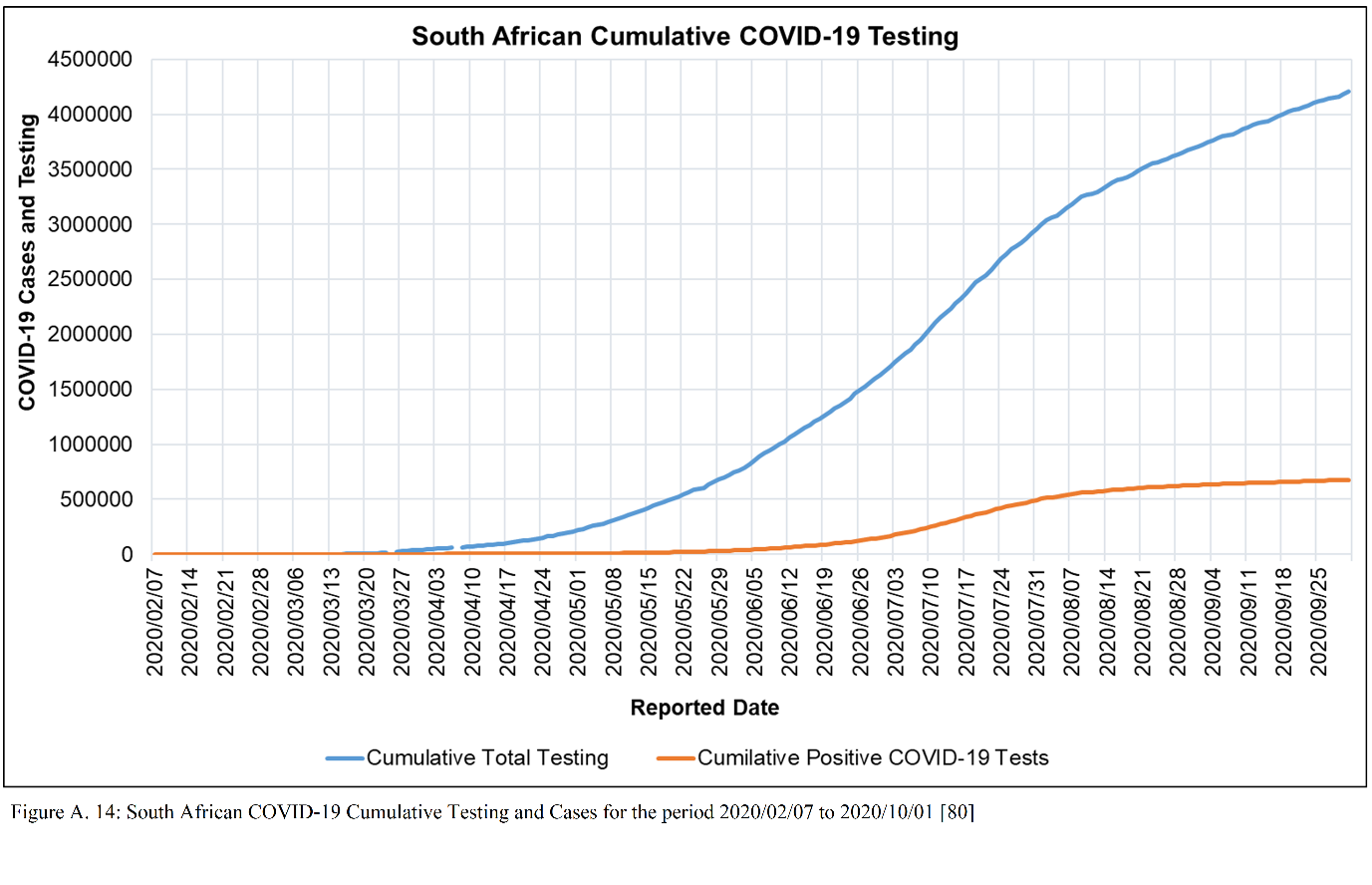


Figure A. 14 South African Cumulative Testing and Cases for the period of 2020/02/07 to 2020/10/01 [80]


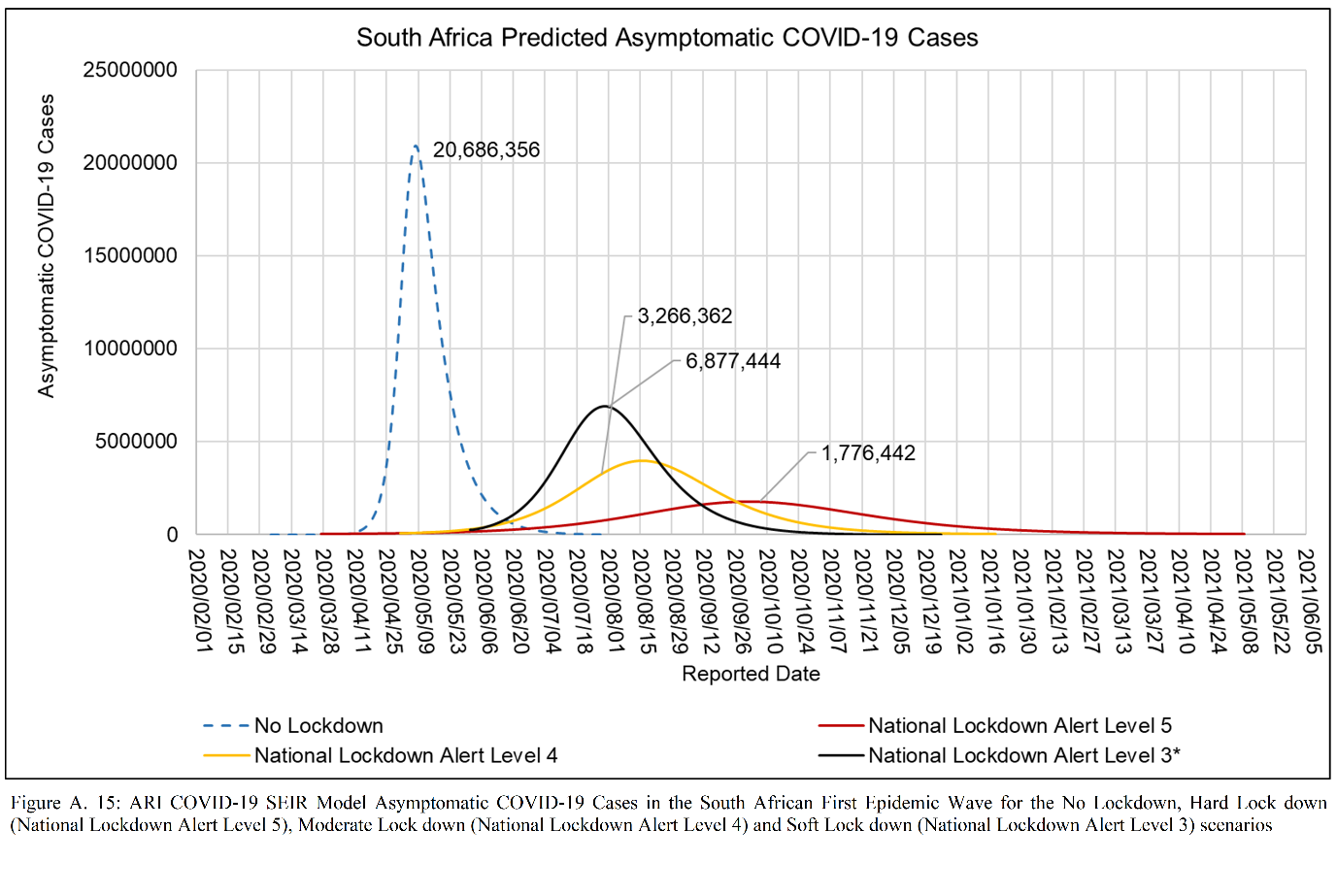


Figure A. 15: ARI COVID-19 SEIR Model Asymptomatic COVID-19 Cases in the South African First Epidemic Wave for the No Lockdown, Hard Lock down (National Lockdown Alert Level 5), Moderate Lock down (National Lockdown Alert Level 4) and Soft Lock down (National Lockdown Alert Level 3) scenarios.
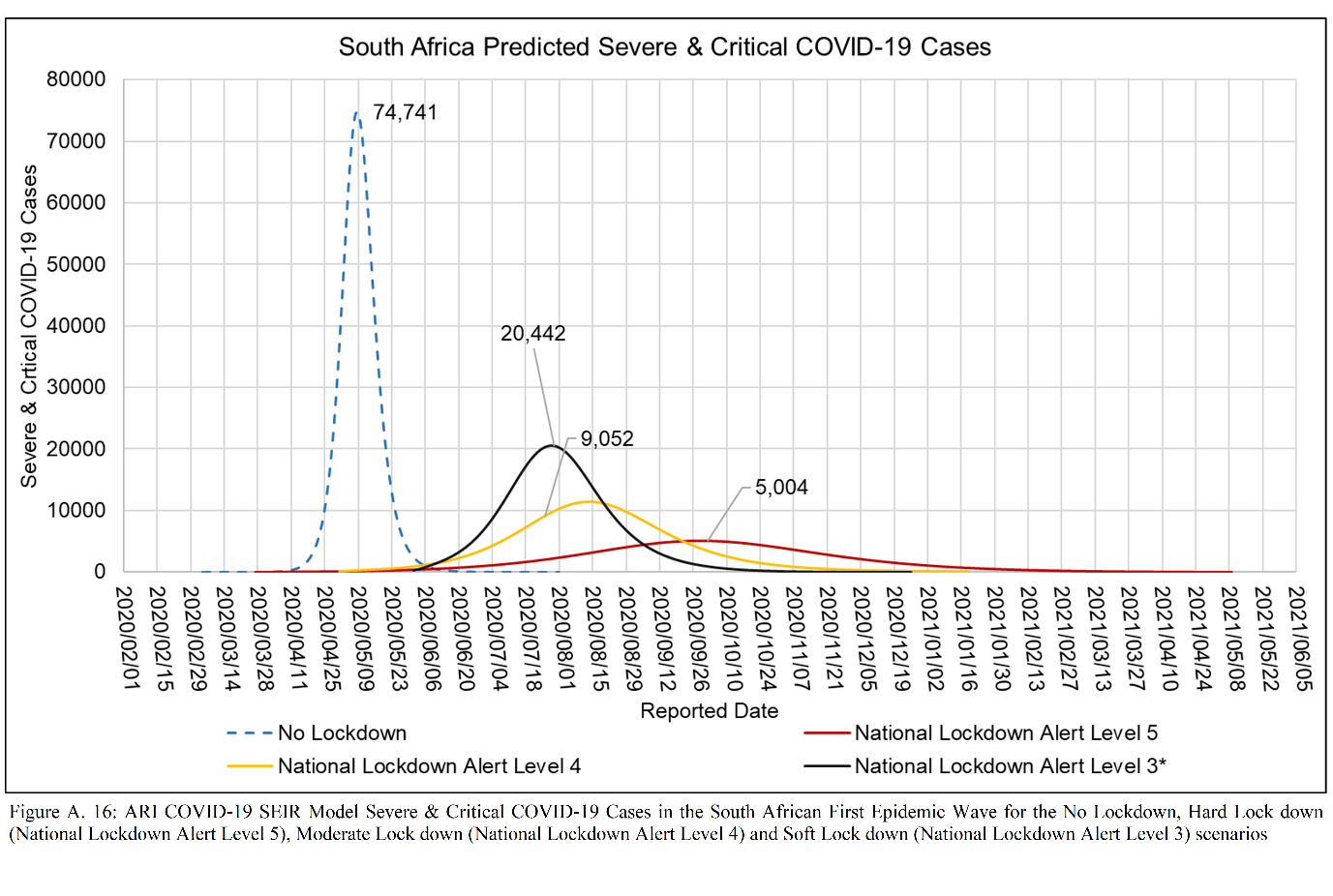


Figure A. 16: ARI COVID-19 SEIR Model Severe & Critical COVID-19 Cases in the South African First Epidemic Wave for the No Lockdown, Hard Lock down (National Lockdown Alert Level 5), Moderate Lock down (National Lockdown Alert Level 4) and Soft Lock down (National Lockdown Alert Level 3) scenarios


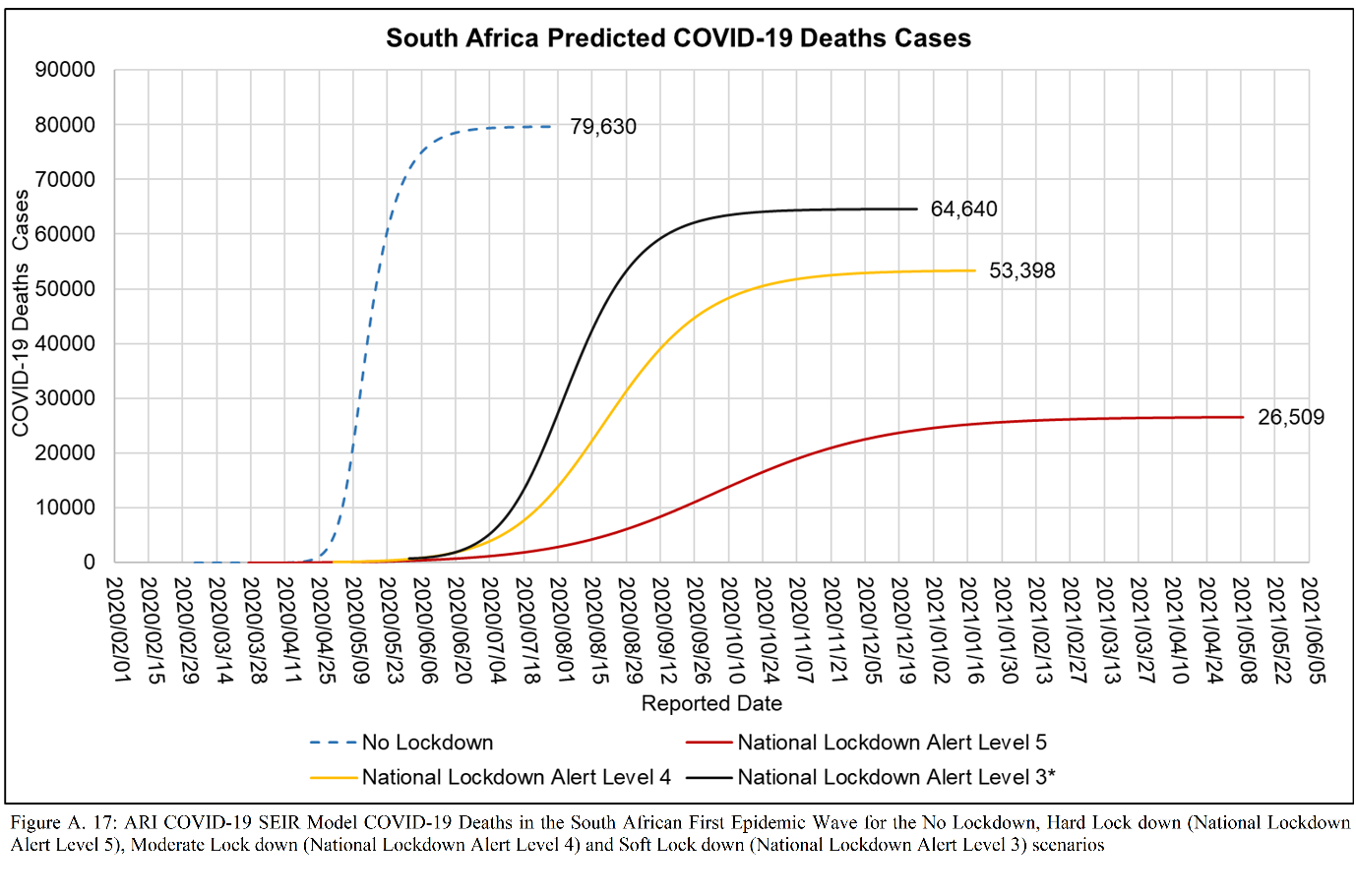


Figure A. 17: ARI COVID-19 SEIR Model COVID-19 Deaths in the South African First Epidemic Wave for the No Lockdown, Hard Lock down (National Lockdown Alert Level 5), Moderate Lock down (National Lockdown Alert Level 4) and Soft Lock down (National Lockdown Alert Level 3) scenarios
